# Antioxidant rich flavonoids from *Oreocnide integrifolia *enhance glucose uptake and insulin secretion and protects pancreatic β-cells from streptozotocin insult

**DOI:** 10.1186/1472-6882-11-126

**Published:** 2011-12-12

**Authors:** Ansarullah, Bhavna Bharucha, Mitesh Dwivedi, Naresh C Laddha, Rasheedunnisa Begum, Anandwardhan A Hardikar, AV Ramachandran

**Affiliations:** 1 Department of Zoology, Faculty of Science, The M.S. University of Baroda, Vadodara 390002, Gujarat, India; 2 Department of Biochemistry, Faculty of Science, The M. S. University of Baroda, Vadodara 390002, Gujarat, India; 3 Diabetes and Pancreas Biology group, The O'Brien Institute, St.Vincent's Hospital and The University of Melbourne, Melbourne, VIC 3065, Australia

## Abstract

**Background:**

Insulin deficiency is the prime basis of all diabetic manifestations and agents that can bring about insulin secretion would be of pivotal significance for cure of diabetes. To test this hypothesis, we carried out bioactivity guided fractionation of *Oreocnide integrifolia *(Urticaceae); a folklore plant consumed for ameliorating diabetic symptoms using experimental models.

**Methods:**

We carried out bioassay guided fractionation using RINmF and C2C12 cell line for glucose stimulated insulin secretion (GSIS) and glucose uptake potential of fractions. Further, the bioactive fraction was challenged for its GSIS in cultured mouse islets with basal (4.5 mM) and stimulated (16.7 mM) levels of glucose concentrations. The Flavonoid rich fraction (FRF) was exposed to 2 mM streptozotocin stress and the anti-ROS/RNS potential was evaluated. Additionally, the bioactive fraction was assessed for its antidiabetic and anti-apoptotic property *in-vivo *using multidose streptozotocin induced diabetes in BALB/c mice.

**Results:**

The results suggested FRF to be the most active fraction as assessed by GSIS in RINm5F cells and its ability for glucose uptake in C2C12 cells. FRF displayed significant potential in terms of increasing intracellular calcium and cAMP levels even in presence of a phosphodiesterase inhibitor, IBMX in cultured pancreatic islets. FRF depicted a dose-dependent reversal of all the cytotoxic manifestations except peroxynitrite and NO formation when subjected *in-vitro *along with STZ. Further scrutinization of FRF for its *in-vivo *antidiabetic property demonstrated improved glycemic indices and decreased pancreatic β-cell apoptosis.

**Conclusions:**

Overall, the flavonoid mixture has shown to have significant insulin secretogogue, insulinomimetic and cytoprotective effects and can be evaluated for clinical trials as a therapeutant in the management of diabetic manifestations.

## Background

Despite being one of the oldest diseases, a composite therapy has eluded the world to date for diabetes mellitus and, WHO has declared it as a chronic disease. It is proving to be a serious life threatening heterogeneous metabolic disorder affecting carbohydrate, lipid and protein metabolisms and afflicting nearly an estimated 191 million people in 2000 and expected to affect an estimated 306 million by 2030 [1,2]. The metabolic derangements characteristic of diabetes in general is primarily a consequence of relative insufficiency of insulin secretion and/or insulin action [3]. Escalating frequency of the disorder is likely to affect developing countries due to expensive and inadequate treatments coupled with the lack of effective and affordable interventions [4,5].

Since the causes of diabetes are multifactorial and fraught with life threatening consequences; the disorder is in urgent need of a multimodal cost-effective therapeutic intervention that is more potent and sans side effects. It is in this context that the World Health Organization (WHO) has encouraged and recommended the use of herbs as an alternative therapy for diabetes [6] and, though a wide range of medicinal plants are in use world over, many of them are however with no valid scientific sanctity. The suggested need for alternative therapy has pinned renewed attention on the search for plant based anti-diabetic agents, also favored due to their easy availability, effectiveness, affordability and probable low ill effects [7]. Apparently, a systematic scientific scrutiny of the anti-diabetic potentials of these plants has become a matter of utmost importance to justify their application in ethnomedicine. Due to their rich diversity and complement of active phytochemicals and secondary metabolites, plants from ancient human civilization have been used as medicament for very many ailments [8-11]. Plants/herbs constitute the main stay of health care system in rural areas due to limited access to modern health facilities.

Indian rural and folklore ethnomedical practices involve usage of many relatively unknown medicinal plants with scientifically non-characterized pharmacological activities. One such folklore plant is "*Oreocnide integrifolia*" popular in northeastern parts of India, especially Manipur, which is consumed as an anti-diabetic therapeutant by Garo, Khasi and Jayantia tribes and by local populace. In this context, we initiated a scientific evaluation of this botanical on various animal models of diabetes [12-14].

Since the primary defects of diabetes centre around β-cell dysfunction, insulin secretion and insulin action, we carried out bioactivity guided assays to identify bioactive fraction using RINm5F and C2C12 cell line as experimental *in-vitro *models for their insulin secretion ability in presence of glucose and their glucose uptake ability in presence of insulin respectively. Flavonoid rich fraction (FRF) exhibited maximal potential in terms of these bioactivities. Further, streptozotocin exposed islets were challenged with FRF to assess glucose stimulated insulin secretion and its cytoprotective potential. Additionally, the dose-dependent anti-diabetic potential of the active fraction (FRF) was also tested *in-vivo *using multidose streptozotocin diabetic mice.

## Methods

### Extraction and fractionation

Fresh green leaves were dried and powdered in a grinder. The powder (100 g) mixed with 500 mL of n-hexane in round bottomed flask was filtered after 48 hrs and the hexane concentrated using a rota evaporator (Buchi, Germany) to obtain hexane fraction. The defatted powder dried free of hexane was subjected to stepwise sequential solvent extraction with Chloroform, Ethyl acetate, Methanol and n-Butanol in a soxhlet apparatus.

### Flavonoid rich fraction

Briefly, One hundred g of air-dried leaves were ground to fine powder and soaked in 70% ethanol for 24 h with continuous stirring. The soaked mixture was filtered using Whatmann No. 1 filter paper and the pellet discarded after centrifugation of the filtrate at 10,000 rpm at room temperature (25°C). The supernatant was concentrated in vacuo by means of a rotavapor and then dissolved in as little water as possible and washed three times with chloroform. The resultant residual layer after extraction three times with ethyl acetate and subjected to concentration *in vacuo *served as the flavonoid rich fraction (FRF).

### *In vitro *and *in vivo *bioactivity assays

#### Mouse Islet Isolation

Islet isolation was as per the methods of [15] and [16]. Briefly, Splenic lobe of pancreas was removed under sterile conditions from groups of three BALB/c mice killed by cervical dislocation without ductal injection and distention. Briefly, the pancreas was cut into small pieces/chopped finely ~1 mm^2 ^and was subjected to enzymatic digestion for 10-12 min by vigorous mechanical shaking in a water bath maintained at 37°C. The dissociation medium consisted of Dulbecco's Modified Minimum Essential Medium (DMEM) supplemented with Collagenase type V (1 mg/mL; Sigma, St. Louis, MO), and 2% BSA fraction V (Hi-Media Labs, Mumbai). The digested tissue was then centrifuged at 1500 g for 10 min, washed twice in PBS (pH: 7.4) and seeded in culture flasks (25 cm^2^; Nunc, Denmark) containing RPMI-1640 (Hyclone, USA) supplemented with 10% (v/v) FBS (Hi-Media, India), 100 U/mL penicillin and 100 U/mL streptomycin under 95% O_2 _and 5% CO_2 _atmosphere at 37°C (Thermo, USA) in air. Under these culture conditions, most of the acinar cells degenerate within 48 hrs leaving behind islets. After 48 hrs of incubation, islets handpicked from exocrine pancreas using a binocular stereomicroscope were quantified using automated Microsoft Excel sheet and number of islet equivalents recorded based on their size. For further purification, islets (freed of acinar cells) were isolated using density gradient Ficoll reagent- Type 400 (Hi-Media Labs, Mumbai) present in 20-11% interface. The islets specificity was assessed using dithizone staining (Hi-Media, Mumbai, Maharashtra, India) while islet viability was performed using Trypan blue staining.

#### Cell lines

RINm5F and C2C12 cell lines procured from National Centre for Cell Sciences, Pune, India served as the experimental cell lines. The RINm5F cells were maintained in RPMI 1640 supplemented with 10% FCS, 100 IU penicillin/mL and 100 μg streptomycin/mL and incubated at 37°C under a humidified 5% CO_2 _atmosphere. The cells allowed to grow in 25 cm^2 ^tissue culture flasks until confluence were then sub-cultured for experimentation. RINm5F cells were subjected to glucose stimulated insulin secretion and cultured in either basal (4.5 mM) or stimulated (16.7 mM) glucose concentrations. Insulin secretion assays were quantified using Mouse/Rat Insulin ELISA kit (Mercodia, Sweden).

#### Glucose induced insulin secretion assay (GSIS)

Isolated islets were cultured at 37°C in a humidified atmosphere of 5% CO_2 _in air in RPMI-1640 medium containing 10% FBS and antibiotics. Islets were seeded at a concentration of 100 or 50 islets per well in 24-well plates (Falcon, NJ) and allowed to attach overnight prior to acute tests. Wells were washed three times with Krebs-Ringer bicarbonate buffer (KRB; 115 mM NaCl, 4.7 mM KCl; 1.3 mM CaCl_2_, 1.2 mM KH_2_PO_4_, 1.2 mM MgSO_4_, 24 mM NaHCO_3_, 10 mM HEPES, 1 g/L BSA, 1.1 mM glucose; pH 7.4) and pre-incubated for 1 hr at 37°C. Unless otherwise stated, wells were then incubated for 1 h in 1 mL KRB with 4.5 mM or 16.7 mM glucose and OI extract/FRF (10, 50, 100 or 250 μg/mL). Aliquots were removed from each well, centrifuged (2000 rpm for 5 min, at 4°C), and assayed for insulin with mouse insulin ELISA kit and protein concentration was determined.

#### 2NBDG glucose uptake

Glucose uptake studies was carried out using fluorescent probe 2-[N-(7-Nitrobenz-2-oxa-1, 3-diazol-4-yl) amino]- 2-deoxy-d-glucose (2NBDG) [17]. Briefly, Dulbecco's Modified Eagle Medium (DMEM) supplemented with 15% Fetal Calf Serum and antibiotics (Penicillin 100 IU/mL and Streptomycin 100 μg/mL) in 5% CO_2 _at 37°C served as the medium for maintenance of C2C12 skeletal muscle cell line. After attainment of ~70% confluency, cells switched to 2% horse serum for 3 days served the purpose of differentiation. The differentiated myotubes were seeded in 96 well fluorescence plates with BSA (1 mg/mL) and 80 μM fluorescent analogue, 2NBDG [Invitrogen, Carlsbad, CA] in presence of different concentrations of various fractions or FRF for 60 min. For stimulation experiments, 100 nM insulin was added along with fractions or FRF. The cells were washed three times to remove free 2NBDG and, plates were read at excitation wavelength of 485 nm and an emission wavelength of 535 nm using Fluorescent micro plate readers (Molecular Devices, Sunnyvale, USA).

#### Dose optimization (islet studies)

Optimization studies for different concentrations/doses of STZ (1 mM, 2 mM and 5 mM) and for duration of STZ exposure (2 hr, 6 hr, 8 hr and 12 hr) were carried out. Different concentrations of FRF (10, 50, 100 and 250 μg/mL) were tested for STZ insult/stress experiments. STZ (2 mM) with an exposure time of 8 hrs was characterized as the best-optimized schedule for experimental studies. Experimental groups consisted of control and STZ treated islets, pre-treated with FRF for a period of 24 hrs.

#### Intracellular Calcium levels

The intracellular calcium concentration, [Ca^2+^]i, was measured using fura-2AM (Molecular Probes, Invitrogen, USA). Fura-2AM crosses cell membranes and once inside the cell, the acetoxymethyl groups removed by cellular esterases generate fura-2, the fluorescent calcium indicator. Islets were incubated in calcium free HBSS with 5 μM fura-2AM at 37°C for 30 min in shaking water bath. After washing (2×) with calcium free HBSS, islets were suspended in complete HBSS and treated with various concentrations of FRF for 60 min in a shaking water bath. Fluorescence was measured in a spectrofluorimeter (Hitachi 7000, Japan) at an emission wavelength of 500 nm for dual excitation wavelength at 340 and 380 nm. The [Ca^2+^]i was expressed as nmole/50 islet equivalents.

#### cAMP estimation

For cAMP measurements, islets cultured in 24-well plates were exposed to 11.1 mM glucose for 60 min in presence of the phosphodiesterase inhibitor 3-isobutyl-1-methylxanthine (1 mM) [IBMX] and various concentrations of FRF. At the end of incubation, islets were lysed following the manufacturer's instructions, and cAMP quantification was performed using a cAMP direct immunoassay kit (Abcam, USA) and normalized to protein concentrations. Results were expressed as fmol/μg protein.

#### MTT assay

The viability of cultured islets was determined by assaying the reduction of 3-(4, 5-dimethylthiazol-2-yl) - 2, 5-diphenyltetrazolium bromide (MTT) to formazan. Briefly, islets were seeded in 24-well microtiter plates (50 islets per well) and left overnight to adhere before being exposed to different concentrations of STZ and FRF. In each experiment, different concentrations of FRF (10, 50, 100, 250 μg/mL) along with 2 mM STZ were tested in three separate wells and the cytotoxicity assessed from at least three different experiments. After exposure to STZ and FRF, incubation of the wells was carried out in dark at 37°C for 4 h after addition of 50 μl of 5 mM MTT solution. Subsequently, formazan crystals were dissolved in 200 μl of DMSO after the removal of MTT and the absorbance measured at 570 nm using a Microplate Reader (Biotek Instruments, USA).

#### ROS estimation

The intracellular formation of reactive oxygen species (ROS) was measured using 2', 7'-dichlorodihydrofluorescin diacetate (DCFH-DA) (Fluka Chemicals, USA). The non-fluorescent compound DCFH-DA penetrates into the cell and is cleaved by intracellular esterases, resulting in the formation of 2', 7'-dichlorodihydrofluorescin (DCFH), the oxidation of which (due to oxidative stress) generates the fluorescent compound dichlorofluorescein. Thus, the DCF fluorescence represents the rate and quantity of ROS produced. Fifty islets from all treatment groups, incubated in fresh media with 10 μM DCFH-DA at 37°C for 30 min, was washed twice with PBS and the fluorescence measured using a fluorimeter (Hitachi 7000, Japan) with excitation at 495 nm and emission at 538 nm. All values were corrected by subtracting auto fluorescence for respective wells and the results expressed as relative fluorescence units/50 islet equivalents

#### Peroxynitrite estimation

Concentration of peroxynitrite from respective treatment groups was estimated by incubating 50 islets with 10 μM dihydrorhodamine 123 (DHR123) for 30 min. Fluorescence was measured using excitation at 500 nm and emission at 530 nm (Hitachi 7000 Spectrofluorimeter). Auto-fluorescence deducted from total fluorescent count yielded corrected fluorescent intensity, expressed as relative fluorescence units/50 islets equivalents.

#### Nitric oxide estimation

For estimation of the concentration of Nitric oxide from respective treatment groups, 50 islets were incubated with the fluorescent probe DAF-FM (1 μM) for 30 min and then washed with PBS. Fluorescence was measured using excitation at 485 nm and emission at 520 nm (Hitachi 7000 Spectrofluorimeter). Auto-fluorescence was deducted from total fluorescent count for corrected fluorescent intensity and was expressed as relative fluorescence units/50 islets equivalents.

#### Evaluation of Mitochondrial Membrane Potential (Δψ_m_)

Mitochondria are vulnerable targets for various toxicants because of their important role in maintaining cellular integrity and functions. Functional alterations occur in mitochondria due to changes in mitochondrial membrane potential. Disruption of the mitochondrial membrane potential (i.e. depolarization) is one of the earliest indicators of cellular disturbance. Fifty islets were loaded with a cationic fluorescent dye Rh-123. From the respective treatment groups, islets changed to serum free media containing Rh-123 (10 μM) were incubated for 30 min at 37°C. Results were expressed as relative fluorescent units/50 islet equivalents.

#### LPO estimation

Lipid peroxidation was monitored in terms of malonaldehydes formed [18]. Briefly, islets were suspended in 200 μl of 1.15% KCl. To this suspension were added 0.8% TBA, 1% SDS and 20% acetic acid. The reaction mixture was kept at 90°C for 45 min and immediately cooled on ice. The pink colour representative of thiobarbituric acid reactive substances was measured at 532 nm and protein concentration determined. The values were expressed as nmoles of MDA formed/mg protein.

#### Multiple dose Streptozotocin model

For induction of diabetes, BALB/c mice (~30 g) were fasted overnight and injected with 50 mg/kg body weight of streptozotocin dissolved in sodium citrate buffer (pH 4.5) intraperitoneally for five consecutive days to delete β-cells. Mice were provided 10% sucrose solution to prevent sudden hypoglycemia. Mice that exhibited frank diabetes (blood glucose levels > 220 mg/dl after 2 weeks) were considered for the experimental studies. Mice were injected flavonoid rich fraction (FRF) for a period of 28 days at a dose 100, 250 and 500 mg/kg of body weight once daily at 1000 hrs. Mice were fasted overnight prior sacrifice. All experiments were carried out according to the guidelines of the Committee for the Purpose of Control and Supervision of Experiments on Animals, India and approved by the Animal Ethical Committee of Department of Zoology, The M.S. University of Baroda, Vadodara (Approval No.827/ac/04/CPCSEA).

#### Glucose and insulin

Blood glucose was estimated using Glucometer (Roche Diagnostics, USA) and insulin levels were estimated using mouse insulin ELISA (Mercodia, Sweden) with an intra-assay of coefficient variance of > 5%.

#### TUNEL/Insulin immunostaining & confocal microscopy

The Terminal dUTP transferase mediated Nick End Labeling (TUNEL) assay was performed to assess the *in situ *DNA fragmentation in paraffin embedded pancreas sections according to the manufacturer's instructions using APO-BrdU™ TUNEL Assay Kit (Invitrogen, USA). Briefly, animals were sacrificed after treatment period and the excised pancreas preserved in 4% paraformaldehyde. Sections were deparaffinised in xylene, downgraded in alcohol grades (100, 95, 85, 70 and 50%), washed with PBS and fixed in fresh 4% paraformaldehyde (PFD) followed by Proteinase-K treatment. Slides were again fixed in 4% PFD and DNA labeling solution added. Guinea pig anti-insulin (1:100, Abcam, USA) was used to probe insulin. Nuclear staining was achieved by adding DAPI/Rnase-A. Anti-Fade solution (Vectamount, Vector Labs, USA) was used as mountant. The slides were visualized by Laser Scanning Confocal Microscope (LSM 510META, ZEISS, Germany). Apoptotic cells exhibited strong nuclear green fluorescence. Optical slices were taken at ~ 0.8 μm. Laser gains, pinhole setting, and magnification was set identical across samples.

#### Statistics

Statistical evaluation of the data was done by one way ANOVA followed by Bonferroni's Multiple comparison test. The results are expressed as mean ± S.E using Graph Pad Prism version 5.0 for Windows, Graph Pad Software, San Diego, California USA.

## Results

### GSIS of various solvent extracts from RINm5F cell line

**Figure 1:**
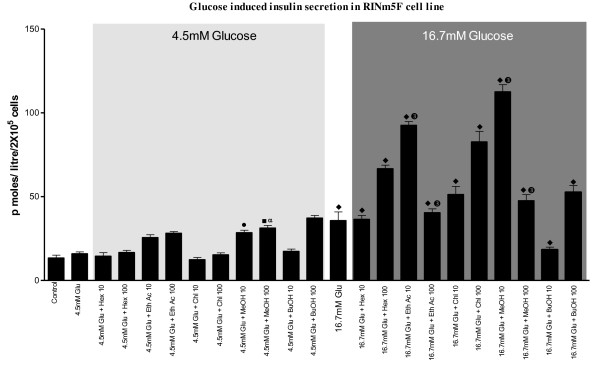
**Effect of different fractions of *Oreocnide integrifolia *on glucose stimulated insulin secretion**. RINm5F cells were either cultured in basal (4.5 mM) or stimulated (16.7 mM) glucose concentrations in presence of fractions. Insulin secretion was quantified using Rat Insulin ELISA kit. Values are expressed as ± SEM in triplicates where • = p < 0.01, ■ = p < 0.001, ♦ = p < 0.0001: when control was compared with rest of the groups; α = p < 0.01, β = p < 0.01, δ = p < 0.01: when 4.5 mM glucose group was compared to rest of the groups and ➊ = p < 0.01, ➋ = p < 0.01, ➌ = p < 0.01: when 16.7 mM glucose group was compared to rest of the groups.

Except for the ethyl acetate and methanol fractions, which showed a dose-dependent increase in insulin release, none of the other fractions showed any noticeable effect on insulin release under basal (4.5 mM glucose) glucose concentration (Figure 1). Under higher concentration of ambient glucose, both ethyl acetate (2-3 fold) and methanol (3-4 fold) fractions showed significant dose-dependent increment in insulin release. In addition, both chloroform and butanol fractions depicted some degree (1.5 fold) of increment in insulin release, especially with the higher dose.

### Promotion of 2NBDG glucose uptake by various solvent extracts in C2C12 cells

**Figure 2:**
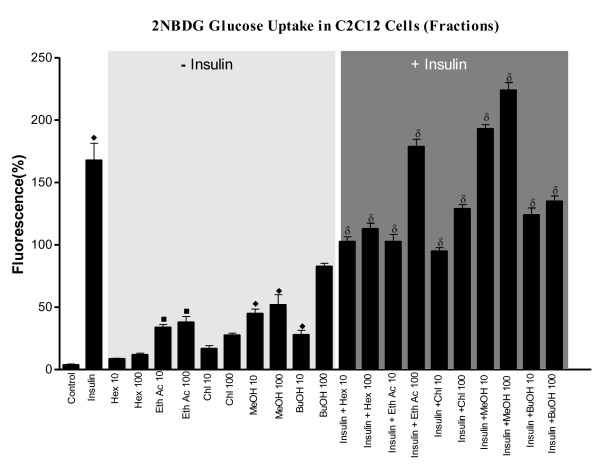
**Effect of different fractions of *Oreocnide integrifolia *on glucose uptake in C2C12 cells**. Differentiated C2C12 cells were incubated with or without 100 nM of insulin along with various fractions. Glucose uptake was measured by using 2NBDG fluorescent probe. Values are expressed as ± SEM in triplicates where • = p < 0.01, ■ = p < 0.001, ♦ = p < 0.0001 when control group was compared with rest of the groups and α = p < 0.01, β = p < 0.01, δ = p < 0.01 when insulin treated group was compared to rest of the groups.

Basal glucose uptake was significantly less in presence of all solvent fractions compared to insulin (Figure 2). However, ethyl acetate and methanol fractions showed dose-dependent increment in glucose uptake, which was significantly greater than other fractions tested. However, higher concentration of n-butanol fraction showed maximal glucose uptake compared to both ethyl acetate and methanol fractions. Even though all fractions showed dose-dependent increment in glucose uptake in presence of insulin, it was still lesser compared to insulin alone, except for the higher dose of ethyl acetate fraction and both doses of methanol fraction, which showed maximal glucose uptake more than that shown by insulin.

### GSIS from RINm5F cells by FRF fraction

**Figure 3:**
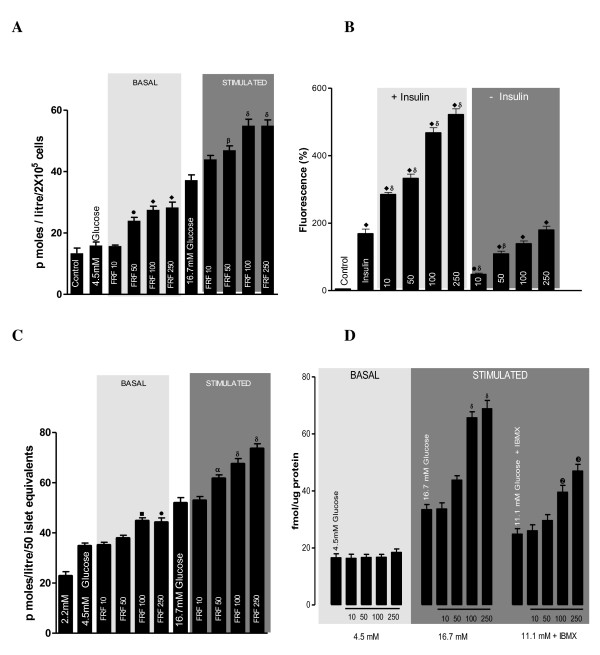
**(A). Represents effect of flavonoidal fraction on glucose stimulated insulin secretion in RINm5F cells**. RINm5F cells were either cultured in basal (4.5 mM) or stimulated (16.7 mM) glucose concentrations in presence of fractions. Insulin secretion was quantified using Rat Insulin ELISA kit. Values are expressed as ± SEM in triplicates where • = p < 0.01, ■ = p < 0.001, ♦ = p < 0.0001 when 4.5 mM glucose group was compared to FRF 10, 50, 100, 250 and α = p < 0.01, β = p < 0.01, δ = p < 0.01 when 16.7 mM glucose group was compared to FRF 10, 50, 100, 250 group.(B) Represents effect of flavonoid fraction on glucose uptake in C2C12 cells. Differentiated C2C12 cells were incubated with or without 100 nM of insulin along with flavonoidal fraction. Glucose uptake was measured by using 2NBDG fluorescent probe. Values are expressed as ± SEM in triplicates where • = p < 0.01, ■ = p < 0.001, ♦ = p < 0.0001 when control was compared with rest of the groups and α = p < 0.01, β = p < 0.01, δ = p < 0.01: Insulin was compared to rest of the groups. (C) Represents effect of flavonoidal fraction on insulin secretion on isolated mouse islets. Islets were either cultured in basal (4.5 mM) or stimulated (16.7 mM) glucose concentrations in presence of flavonoidal fraction. Insulin secretion was quantified using Mouse Insulin ELISA kit. Values are expressed as ± SEM in triplicates where • = p < 0.01, ■ = p < 0.001, ♦ = p < 0.0001 when 4.5 mM glucose group was compared to FRF 10, 50, 100, 250 groups and α = p < 0.01, β = p < 0.01, δ = p < 0.01 when 16.7 mM glucose group was compared to FRF 10, 50, 100, 250. (D) Represents effect of flavonoidal fraction on cAMP levels in mouse islets. Cultured islets were exposed to 11.1 mM for 60 min in the presence of phosphodiesterase inhibitor (1 mM IBMX) along with flavonoidal fraction. Values are expressed as ± SEM in triplicates where α = p < 0.01, β = p < 0.01, δ = p < 0.01 when 16.7 mM glucose group was compared to its 10, 50, 100, 250 groups and ➊ = p < 0.01, ➋ = p < 0.01, ➌ = p < 0.01 when 11.1 mM glucose + IBMX group was compared to its 10, 50, 100, 250 groups.

Under both basal (4.5 mM) and higher (16.7 mM) glucose ambient concentrations, the FRF fraction depicted a dose- dependent increment in insulin secretion with the highest dose showing almost doubled insulin secretion (Figure 3A).

### FRF stimulated 2NBDG glucose uptake by C2C12 cells

The FRF fraction depicted a dose-dependent 5-15 fold increase in glucose uptake even in the absence of insulin. The insulin induced glucose uptake was further stimulated 2-5 fold in a dose-dependent manner by the FRF fraction (Figure 3B).

### GSIS by FRF from isolated mouse islets

The FRF induced significant dose-dependent increment in insulin secretion from mouse islets under both basal (4.5 mM) and stimulated (16.7 mM) glucose concentrations (Figure 3C). The increment in insulin release under glucose challenge was significantly greater than in the basal state. The highest dose of FRF showed near 45% increase in GSIS.

### Effect of FRF on glucose induced increase in cAMP and Ca^++ ^levels in islets

**Figure 4:**
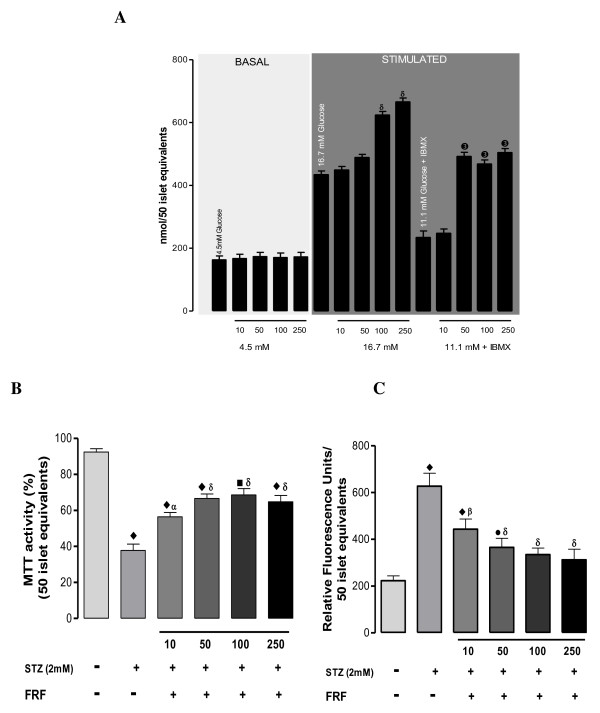
**(A) Represents effect of flavonoidal fraction on intracellular calcium levels in mouse islets**. Intracellular calcium levels were measured using fluorescent probe fura-2AM. Values are expressed as ± SEM in triplicates where α = p < 0.01, β = p < 0.01, δ = p < 0.01 when 16.7 mM glucose group was compared to its 10, 50, 100, 250 groups and ➊ = p < 0.01, ➋ = p < 0.01, ➌ = p < 0.01 when 11.1 mM glucose + IBMX group was compared to its 10, 50, 100, 250 groups. (B) Represents effect of flavonoidal fraction on MTT activity in mouse islets. Cultured islets were exposed to pancreatic β- cell specific toxin, streptozotocin (2 mM) along with flavonoidal fraction at various concentrations and assessed for cytotoxicity. Values are expressed as ± SEM in triplicates where • = p < 0.01, ■ = p < 0.001, ♦ = p < 0.0001 when control group was compared with rest of the groups and α = p < 0.01, β = p < 0.01, δ = p < 0.01 where diabetic group was compared to rest of the groups. (C) Represents effect of flavonoidal fraction on intracellular ROS activity in mouse islets. Intracellular ROS was assessed by fluorescent probe DCFH-DA. Values are expressed as ± SEM in triplicates where • = p < 0.01, ■ = p < 0.001, ♦ = p < 0.0001 when control group was compared with rest of the groups and α = p < 0.01, β = p < 0.01, δ = p < 0.01 when diabetic group was compared to rest of the groups.

There was no increase in intra-islet cAMP (Figure 3D) or Ca^++ ^(Figure 4A) on exposure to any of the doses of FRF under basal ambient glucose level. However, under stimulating concentration of glucose, a dose-dependent increment by FRF in intra islet cAMP and Ca^++ ^levels could be noticed. Further, this dose-dependent effect of FRF on cAMP and Ca^++ ^levels could be discerned in a medium containing less stimulating concentration (11.1 mM) of glucose and the PDE inhibitor IBMX.

### *In-vitro *assessment of the effect of FRF on streptozotocin induced islet viability and oxidative stress

**Figure 5:**
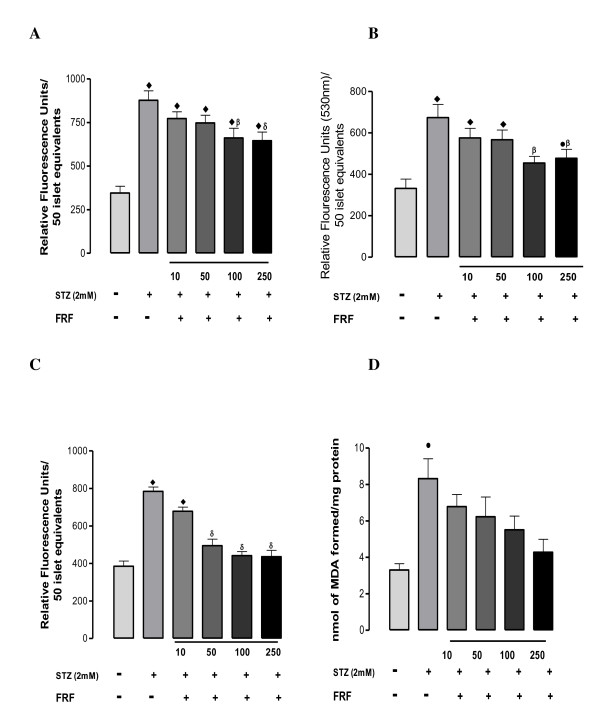
**(A) Represents effect of flavonoidal fraction on peroxynitrite levels in mouse islets exposed to streptozotocin**. Peroxynitrite levels were quantified by fluorescent probe dihydrorhodamine 123. Values are expressed as ± SEM in triplicates where • = p < 0.01, ■ = p < 0.001, ♦ = p < 0.0001 when control group was compared with rest of the groups and α = p < 0.01, β = p < 0.01, δ = p < 0.01 when diabetic group was compared to rest of the groups (B) Represents effect of flavonoidal fraction on NO levels in mouse islets exposed to streptozotocin. Nitric oxide levels were quantified by fluorescent probe DAF-FM. Values are expressed as ± SEM in triplicates where • = p < 0.01, ■ = p < 0.001, ♦ = p < 0.0001 when control group was compared with rest of the groups and α = p < 0.01, β = p < 0.01, δ = p < 0.01 when diabetic group was compared to rest of the groups (C) Represents effect of flavonoidal fraction on mitochondrial membrane potential (MMP) in mouse islets exposed to streptozotocin. MMP levels were quantified by fluorescent probe Rhodamine 123. Values are expressed as ± SEM in triplicates where • = p < 0.01, ■ = p < 0.001, ♦ = p < 0.0001 when control group was compared with rest of the groups and α = p < 0.01, β = p < 0.01, δ = p < 0.01 when diabetic group was compared to rest of the groups (D) Represents effect of flavonoidal fraction on lipid peroxidation levels (LPO) in mouse islets exposed to streptozotocin. Lipid peroxidation was measured by using thiobarbituric acid reagent. Values are expressed as ± SEM in triplicates where • = p < 0.01, ■ = p < 0.001, ♦ = p < 0.0001 when control group was compared with rest of the groups.

All oxidative stress parameters, MTT (Figure 4B), DCF (Figure 4C), Peroxynitrite (Figure 5A), NO (Figure 5B), Rh 123 (Figure 5C), and LPO (Figure 5D), were all significantly elevated in presence of streptozotocin. FRF protected the islets against STZ induced oxidative stress in a dose-dependent manner with the highest dose affording near total protection. Streptozotocin significantly reduced cell viability, which was protected against by the co-presence of FRF in a dose-dependent mode.

### Assessment of the anti-diabetic potential of FRF in multidose STZ mice

#### Blood glucose and insulin level

At the end of 28 day period streptozotocin diabetic mice displayed significant hyperglycemia and hypoinsulinemia. FRF treated mice showed significant increase in plasma insulin and concomitant decrease in glucose in a dose-dependent manner (Table 1).

**Table 1:** Effect of FRF on plasma glucose levels and insulin titres in normoglycaemic and mSTZ diabetic mice.

	Plasma Glucose (mg/dl)	Plasma insulin (μIU/ml)
Control	107.67 ± 4.50	22.64 ± 0.75

Diabetic	249.50 ± 8.97^♦^	12.76 ± 1.02^♦^

Diabetic + FRF 100	180.76 ± 7.76^♦ **δ**^	13.65 ± 0.76^♦^

Diabetic + FRF 250	167.60 ± 8.30**^δ^**	17.86 ± 0.78^•**β**^

Diabetic + FRF 500	163.98 ± 7.22**^δ^**	17.92 ± 0.72^•**α**^

All values are expressed as ± SEM (n = 6) where • = p < 0.01, ■ = p < 0.001, ♦ = p < 0.0001 when control group was compared with rest of the groups and α = p < 0.01, β = p < 0.01, δ = p < 0.01 when diabetic group was compared with FRF 100, 250, 500 groups

#### Histopathological observations

**Figure 6:**
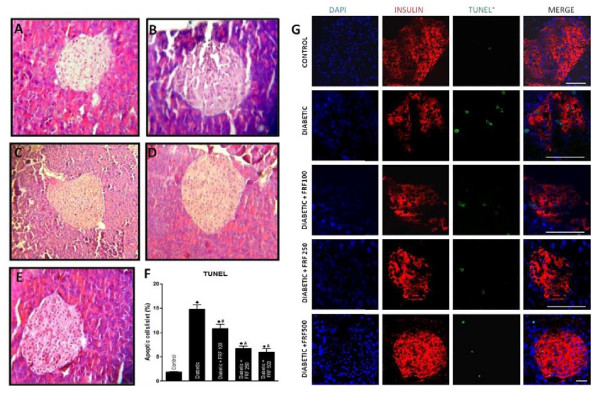
**Images represent haemetoxylin and eosin stained pancreas sections of (A) control mice showing intact islet histoarchitecture, (B) diabetic mice showing islet cell destruction and wider intracellular spaces due to streptozotocin toxicity (C-E) diabetic animals treated with flavonoidal fraction at 100, 250 and 500 mg/kg bodyweight demonstrating ameliorating effects and improved islet integrity (Magnification 200×) (F) Quantification of islet cell apoptosis in multiple dose streptozotocin mouse treated with various concentrations of flavonoidal fraction (G) Images represent confocal optical slices of immunostained pancreas of control, diabetic and diabetic treated with various concentrations of flavonoidal fraction**. Guinea Pig anti insulin (red) and ApoBrdu-dUTP (green) were used as primary antibodies. DAPI (Blue) was used to visualize nuclei. The slides were visualized by Laser Scanning Confocal Microscope (LSM 510 META, ZEISS, Germany). Scale bar represents 100 μm).

The pancreatic islets of streptozotocin-administered mice depicted β-cell loss and wider intracellular spaces due to β-cell toxicity of STZ (Figure 6A-E). FRF supplementation showed recovery of the structural integrity of islets with the pancreas of mice administered highest dose of FRF depicting near normal islet histoarchitecture.

#### Immunostaining of pancreas for cellular apoptosis and insulin localization

The pancreatic islets of STZ treated mice depicted greater number of TUNEL positive cells and on quantitative terms a six fold higher percentage of apoptotic cells (Figure 6F). Pancreatic islets of STZ treated mice demonstrated reduced immunoreactivity for insulin while, the islets of FRF supplemented mice showed increased insulin immunoreactivity, with 250 μg FRF treated islets showing strong immunoreactivity (Figure 6G).

## Discussion

The various extracts tested for their GSIS from RINm5F cells clearly show stimulatory effects for only ethanolic and methanolic extracts under basal glucose concentration of 4.5 mM. Both these extracts show augmented GSIS under a stimulating glucose concentration of 16.7 mM glucose with the effect of methanolic extract being maximal. However, 100 μg/mL butanol fraction also shows a significant GSIS. Apparently, active phytochemical ingredients with insulin secretogogue properties seem to be present in alcoholic extracts of OI, suggesting the secretogogues to be polar in nature. The herein inferred importance of polar compounds, extractable in alcoholic solvents, in inducing GSIS finds support in the recent reports of alcoholic extracts of many plants showing potential for glucose stimulated insulin secretion from cell lines [19,20]. Some of the active polar compounds also seem to possess insulinomimetic action as seen by the ability of alcoholic extracts to promote glucose uptake in C2C12 Cells. Though these fractions promoted glucose uptake, it however was relatively lesser in comparison to insulin. Even in presence of insulin, though hexane, ethanolic and butanol fractions showed enhanced glucose uptake more than that seen without insulin, was nevertheless still lesser than insulin. However, methanolic fraction shows a complementary effect with insulin as, both doses of the extract promoted greater glucose uptake than insulin alone. Apparently, the methanolic extract has the active principle(s) that promote(s) glucose transport in larger concentration than in other fractions. The possible reason for the observed attenuation of glucose uptake (transport) promoting activity of the alcoholic extracts compared to insulin could be the presence of other interfering substances in the crude extracts that reduce the potential of the promoting molecules. Presence of phytochemicals in alcoholic extracts of other plants, which promote glucose uptake in muscle and adipose cell lines [21-23], substantiate our observed effect.

Based on our observations of methanolic fraction being more active in terms of insulin secretogogue and insulinomimetic functions and, richer flavonoid content, the isolated FRF was tested for its GSIS and glucose uptake properties. The observations made on RINm5F and C2C12 cell lines clearly establish the very potent ability for eliciting insulin release and glucose uptake. The very high potency of our FRF fraction for glucoregulation is indicated by the dose-dependent tremendously high insulin secretogogue expression (46% to 86% under basal glucose concentration and 11% to 37% under stimulating glucose concentration) and insulinomimetic role in glucose uptake (83% to 240% in the absence of insulin and 250% to 1450% in presence of insulin). Relatively, promotion of glucose uptake seems to be the more significant function of FRF than, elicitation of insulin release. The observed markedly high potentials of FRF can be related with the identified mixture of flavonoids functioning together, either in an additive or synergistic fashion. Though there are reports of flavonoids promoting insulin secretion and glucose uptake under *in vitro *conditions [24-26], our FRF seems to exert relatively more powerful effects. The *in vitro *effect of FRF on insulin secretion seen in cell lines stands confirmed by the observed dose-dependent increment by 23% under basal and by 44% under stimulated conditions of insulin release from isolated whole mouse islets. Clearly, the FRF mixture of OI has appreciable insulin secretogogue ability and is ideal for treatment of insulin deficiency under diabetic state.

The mechanism of glucose induced insulin secretion and other insulinotropic augmenters acting along with glucose, bring about ATP mediated closure of membrane K^+ ^channels and consequent depolarization leading to opening of Ca^++ ^channels and intra-cellular elevation in Ca^++ ^that mediate insulin secretory vesicle exocytosis. Most of the insulinotropic agents or potentiators function in a back ground of glucose stimulated permissive environment and stimulate adenylate cyclase mediated cAMP production and PLC-β mediated formation IP3 and DAG resulting in intracellular Ca^++ ^signalling that culminate in PKA and PKC activation, which could in turn phosphorylate and again activate K_ATP _channels. Elevation of these second messengers can also enhance insulin gene transcription [27,28]. Even other pathways of insulin release independent of exocytosis, by altered membrane permeability stand reported [29,30]. Presently, the FRF fraction elevated the intra islet levels of both cAMP and Ca^++ ^in a dose dependent manner in presence of stimulating concentration of glucose. The dose-dependent increase in cAMP is manifested even in presence of IBMX, the PDE inhibitor. All the three doses of FRF equally increase Ca^++ ^level in presence of IBMX. It would appear from the present observations that, our FRF has potent ability to function as a potentiating insulinotropic agent for stimulating cAMP and Ca^++ ^mediated insulin secretion under the permissive influence of glucose. It is likely that, FRF may also mediate its insulin secretogogue function through GLP-1 action as, flavonoids are known to increase GLP-1 level as well as inhibit dipeptidyl peptidase IV (DPP-IV), the enzyme that metabolizes GLP-1, both of which can efficiently up regulate insulin secretion from pancreatic islets in presence of glucose [31,32].

Exposure of islets to STZ shows significantly increased formation of peroxynitrite, NO and ROS with markedly elevated LPO and reduced cell viability. Islets exposed to STZ also show significantly increased mitochondrial membrane potential. Apparently, STZ causes severe oxidative and cytotoxic stress to islets that is likely to compromise their insulin releasing capacity. Presence of FRF in the medium along with STZ has depicted a dose dependent reversal of all the cytotoxic manifestations, with the highest dose of 250 μg/mL normalizing most of the measured parameters except for peroxynitrite and NO formation and total cell viability. This suggests the need for a probably higher dose to completely nullify the NO and peroxynitrite generation potential of STZ. The herein observed anti-oxidant, anti-ROS and NOs generation potentials and, maintenance of mitochondrial membrane potential find substantial support from the reported anti-oxidant potential of flavonoids [33-35].

Validation of observed *in vitro *effects of FRF in multidose STZ mice has provided strong supportive evidence as, FRF has shown a dose-dependent decrease in blood glucose level and increase in plasma insulin titer. Cytoprotective effect of FRF is also well evidenced by the histological appearance of islets from STZ+FRF mice compared to STZ mice. Further proof for the cytoprotective effect of FRF is provided by its anti-apoptotic potential as seen from the TUNEL assay and the noticeable insulin immunoreactivity. Many medicinal plants have been shown to have significant antidiabetic effects suggesting the presence of active phytochemicals [36-39].

## Conclusions

Overall, the present investigation has shown the presence of active phytochemicals in the methanolic extract of OI and a rich mix of flavonoids. Hence, the present study indicates the therapeutic potential of flavonoid rich fraction to have significant insulin secretogogue, insulinomimetic and cytoprotective effects. We recently demonstrated the role of *Oreocnide *flavonoids in pancreatic beta cell regeneration mouse model [40]. Further, clinical trials are necessary to assess the therapeutic potential of flavonoid rich fraction to counteract the manifestations of diabetes mellitus.

## Competing interests

All authors ANS, BB, MD, NCL, RB, AAH, AVR declare that they have no competing interest

## Authors' contributions

ANS, BB, MD and NCL performed *in-vitro *and *in-vivo *analysis while RB, AAH and AVR designed, reviewed and interpreted the data. All authors read and approved the manuscript.

## Pre-publication history

The pre-publication history for this paper can be accessed here:

http://www.biomedcentral.com/1472-6882/11/126/prepub
